# PTEN inhibitor VO-OHpic protects endplate chondrocytes against apoptosis and calcification via activating Nrf-2 signaling pathway

**DOI:** 10.18632/aging.204612

**Published:** 2023-03-24

**Authors:** Xingang Cui, Xiaoyang Liu, Peng Kong, Ting Du, Tao Li, Guihe Yang, Weimin Zhang, Xingzhi Jing, Wenchao Wang

**Affiliations:** 1 Department of Spine Surgery, Shandong Provincial Hospital, Shandong University, Jinan, Shandong 250000, China; 2 Department of Spine Surgery, Shandong Provincial Hospital Affiliated to Shandong First Medical University, Jinan, Shandong 250000, China; 3 Department of Orthopaedics, The Affiliated Hospital of Shandong University of Traditional Chinese Medicine, Jinan, Shandong 250000, China; 4 Department of Medical, Yidu Cloud (Beijing) Technology Co. Ltd., Beijing 100191, China

**Keywords:** PTEN inhibitor, cartilage endplate, Nrf-2, mitophagy, ferroptosis

## Abstract

Cartilage endplate (CEP) degeneration and calcification is an important contributor to the onset and pathogenesis of intervertebral disc degeneration (IDD). However, the underlying mechanisms of CEP degeneration remain elusive, let alone according treatment strategies to prevent CEP degeneration. Phosphatase and tensin homolog (PTEN) is a tumor suppressor gene that promotes cell apoptosis, and recent studies indicated that PTEN is overexpressed in degenerated intervertebral disc. However, whether direct inhibition of PTEN attenuates CEP degeneration and IDD development remains largely unknown. In the present study, our *in vivo* experiments demonstrated that VO-OHpic could attenuate IDD progression and CEP calcification. We also found that VO-OHpic inhibited oxidative stress induced chondrocytes apoptosis and degeneration by activating Nrf-2/HO-1 pathway, thus promoted parkin mediated mitophagy process and inhibited chondrocytes ferroptosis, alleviated redox imbalance and eventually improved cell survival. Nrf-2 siRNA transfection significantly reversed the protective effect of VO-OHpic on endplate chondrocytes. In conclusion, our study demonstrated that inhibition of PTEN with VO-OHpic attenuates CEP calcification and IDD progression. Moreover, VO-OHpic protects endplate chondrocytes against apoptosis and degeneration via activating Nrf-2/HO-1 mediated mitophagy process and ferroptosis inhibition. Our results suggest that VO-OHpic may be a potential effective medicine for IDD prevention and treatment.

## INTRODUCTION

Intervertebral disc degeneration (IDD) is a major cause of low back pain and affects 80% of the population worldwide, which results in job-related disability and high healthcare costs [1]. Intervertebral disc is the largest avascular tissue which is composed of the nucleus pulposus (NP), the annulus fibrosus (AF), upper and lower cartilage endplate (CEP) [2]. The nutrient and oxygen supply of intervertebral disc is mainly through the penetration of cartilage endplate, CEP calcification and degeneration could significantly reduce the nutrient supply of intervertebral discs and is the leading cause of IDD [3, 4]. However, relatively few studies have focused on the CEP degeneration, let alone according treatment strategies to prevent CEP degeneration.

Phosphatase and tensin homolog (PTEN) is encoded by a 200 kb gene located on chromosome10q23, and is reported to be involved in many diseases [5]. Through converting phosphatidylinositol (3,4,5)-triphosphate (PIP3) to phosphatidylinositol (4,5)-bisphosphate (PIP2), PTEN can counteract the PI3K/AKT signaling activation and is involved in many pathological and physiological processes [6]. The PTEN/PI3K/AKT signaling pathway plays important roles in many diseases including neurodegenerative diseases, cardiovascular diseases and cancers [7]. Recently, PTEN was reported to be overexpressed in degenerated NP cells and is responsible for NP cells apoptosis [8, 9]. Moreover, the essential role of PTEN in osteoarthritis development has recently been clarified, PTEN could decrease the chondrocytes viability and type II collagen production by inhibiting PI3K/AKT activation [10]. However, to our knowledge, no studies have investigated the role of PTEN in CEP degeneration and calcification. VO-OHpic is a reversible non-competitive PTEN inhibitor which is based on vanadium [11]. PTEN inhibition with VO-OHpic has been reported to be able to attenuate cell apoptosis and cardiovascular diseases development [12]. VO-OHpic has been proved safe and highly selective, it could inhibit PTEN with low nanomolar concentrations. Moreover, previous studies have demonstrated that the inhibition effect of PTEN by VO-OHpic is reversible [7]. In the present study, we aim to investigate whether VO-OHpic could inhibit CEP chondrocytes apoptosis and degeneration and whether VO-OHpic could protect against CEP calcification and IDD development.

Reactive oxygen species (ROS) are mainly generated by mitochondria and are involved in numerous signaling pathways, including matrix metalloproteinases (MMPS) expression and cartilage degeneration [13]. Under physiological condition, mitochondrial dysfunction will generate excess ROS and ROS overproduction will cause oxidative stress, leading to mitochondrial dysfunction and resulting in a vicious cycle. Mitochondrial autophagy (Mitophagy) could decrease excess ROS generation and restore redox balance via eliminating damaged mitochondria [14]. Recent articles indicated that parkin, the mitophagy marker, was decreased in degenerated NP, and mitophagy activation could prevent oxidative stress induced NP and cartilage chondrocytes degeneration [15, 16].

Nuclear factor erythroid 2-related factor 2 (Nrf2) is responsible for cellular redox homeostasis and serves as the key sensor of oxidative stress. When activated, Nrf-2 translocates from the cytoplasm to nucleus and activates its downstream antioxidant enzymes, including heme oxygenase (HO-1), NADPH, NQO1 and thioredoxin (Trx) [17]. Accumulating articles have reported the important role of Nrf-2 in osteoarthritis progression and activation of Nrf-2 could mitigate the inflammatory process [18]. Recent studies also found that Nrf-2 activation could slow down the IDD development by inhibiting the oxidative stress and inflammatory process in NP cells [19]. Moreover, recent study demonstrated that ferroptosis plays a role in NP cells viability and Nrf-2 could regulate ferroptosis marker, GPX4 and iron storage proteins ferritin light and heavy chains (FTL/FTH1) [20]. There are studies which also indicate that PI3K/AKT pathway is involved in the Nrf-2 activation and translocation [21]. Consistently, PTEN could significantly repress the activity of Nrf-2, while loss of PTEN could lead to the activation of Nrf-2 [22].

In the present study, we investigated whether inhibition of PTEN with VO-OHpic could ameliorate oxidative stress induced CEP chondrocytes apoptosis and degeneration. Also *in vivo* experiments were conducted to investigate the effects of VO-OHpic in attenuating cartilage endplate calcification and inhibiting IDD development.

## RESULTS

### VO-OHpic significantly ameliorated IDD progression and cartilage endplate calcification *in vivo*

**Figure 1:**
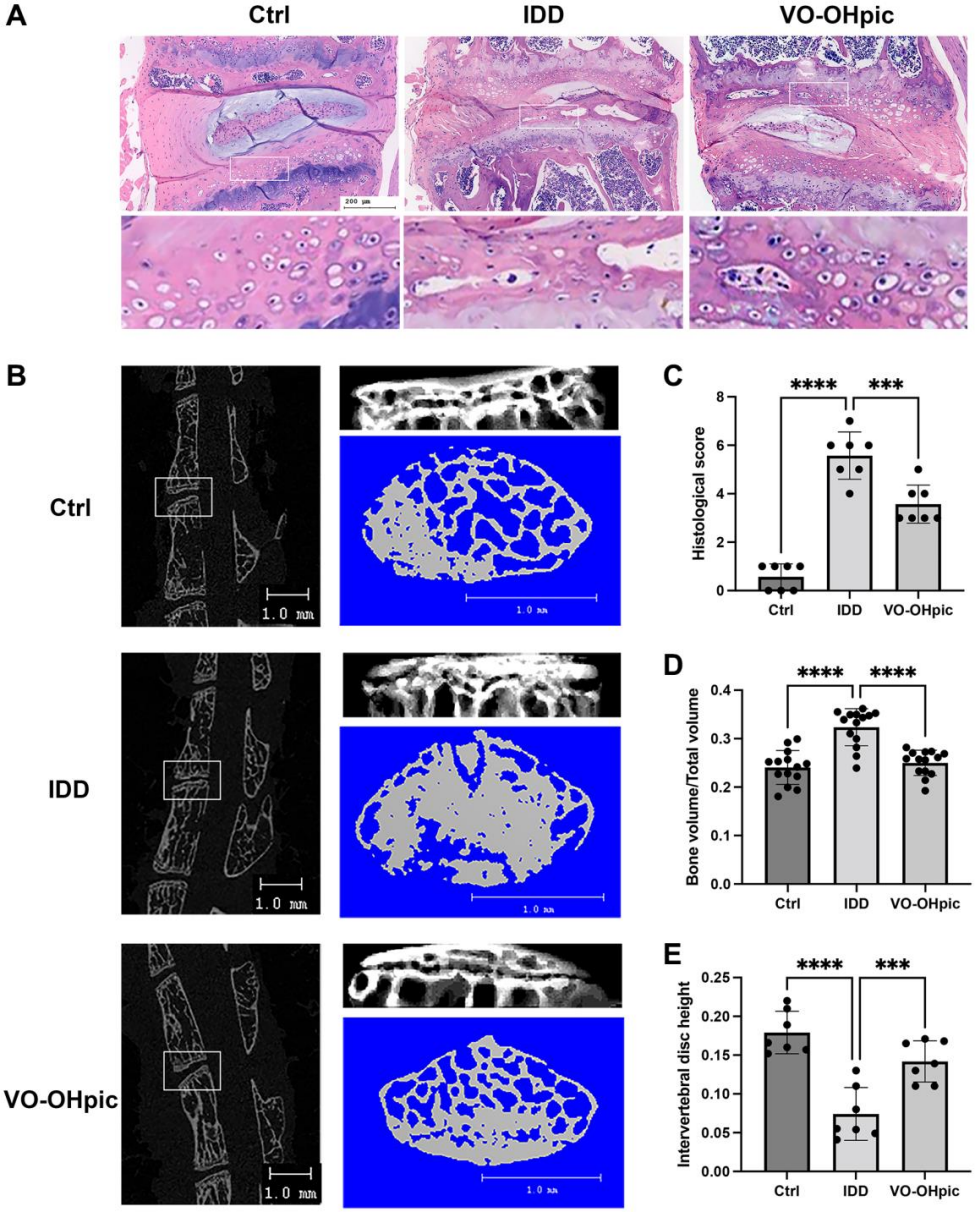
**VO-OHpic significantly ameliorated IDD progression and cartilage endplate calcification *in vivo*.** (**A**) HE staining of L4/5 intervertebral discs in Ctrl group, IDD group and IDD+VO-OHpic group. Scale bar = 200 μm. (**B**) Micro-CT analysis of L4/5 segments and cartilage endplate. Scale bar = 1 mm. (**C**) Histological score of the L4–5 segments of the lumbar spine among the three groups. (**D**) Quantification of cartilage endplate calcification via microarchitecture parameters (bone volume per tissue volume (BV/TV)). (**E**) Histomorphometric assessment of the intervertebral disc height. The disc height was calculated by the average of the anterior, middle and posterior of intervertebral disc. Data are presented as mean ± SD (*n* = 7/group). ^***^P < 0.001, ^****^P < 0.0001.

We established an IDD mice model by transection of the L4/5 bilateral facet joints, supra- and interspinous ligaments to investigate the effects of VO-OHpic in IDD *in vivo*. The IDD degeneration was scored by immunohistochemistry staining and micro-CT analysis. As shown in Figure 1A, immunohistochemistry staining showed that mice in IDD group exhibited reduced NP and obvious cleft in annulus fibrosus. Bone marrow and empty bone and chondrocyte lacuna were found in the deep zone of CEP. Compared to mice in IDD group, the CEP in VO-OHpic treatment group was thicker with much less bony tissues. The AF and NP were intact. These results demonstrated that VO-OHpic could inhibit CEP degeneration and IDD progress. The IDD degeneration score in VO-OHpic group was significantly lower compared to that of IDD group (Figure 1C). Figure 1B showed 3D reconstruction of Micro-CT results of vertebral body and cartilage endplate. As shown in Figure 1D, 1E, quantification of Micro-CT analysis showed significantly decreased intervertebral disc height and increased bone mineral density of CEP in IDD group, and it was significantly improved by VO-OHpic administration. These results indicate that VO-OHpic treatment could ameliorate IDD development and CEP degeneration *in vivo*.

### VO-OHpic exerted protective effect in oxidative stress induced endplate chondrocytes apoptosis and CEP degeneration

**Figure 2:**
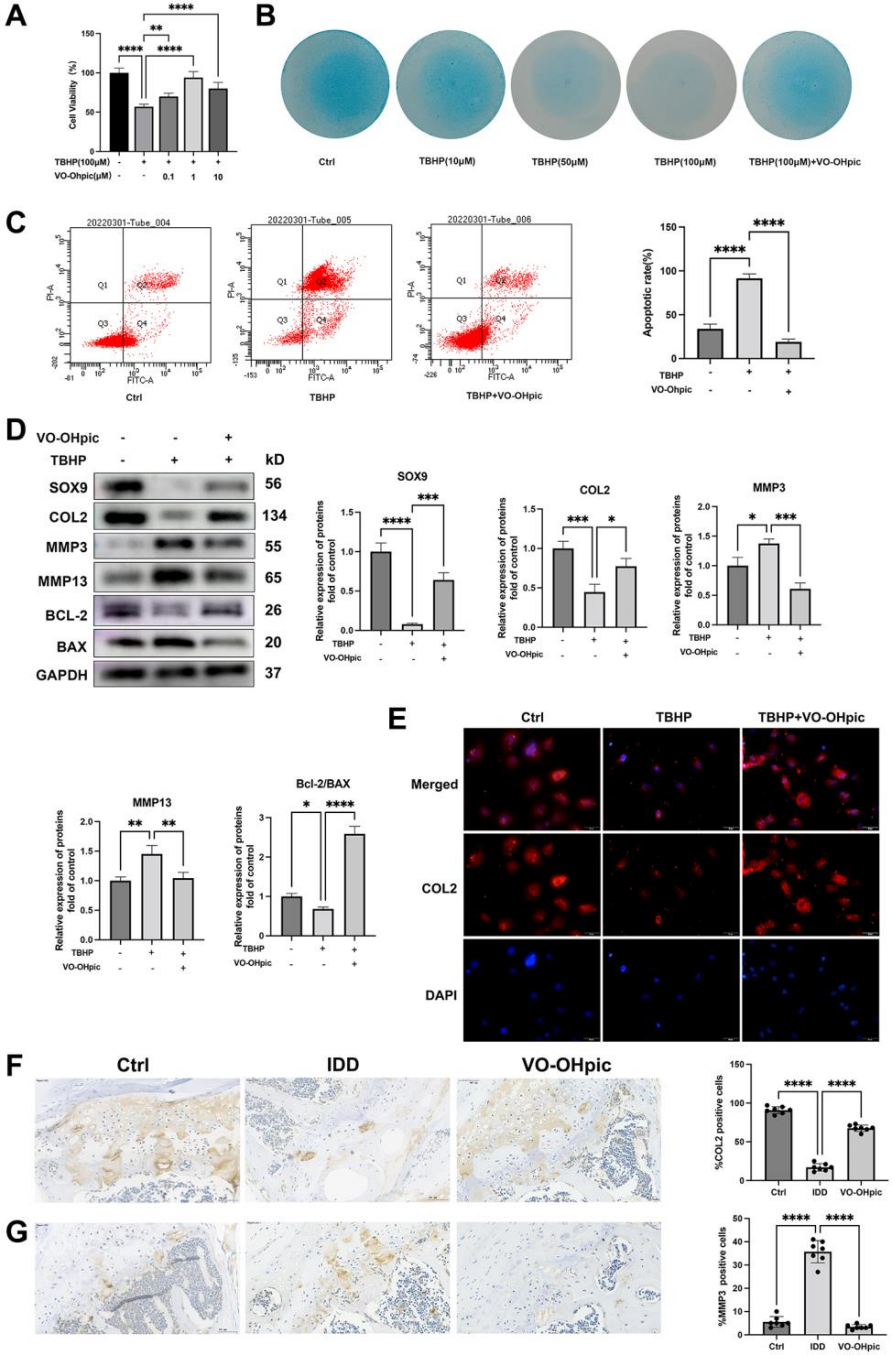
**VO-OHpic inhibited oxidative stress induced endplate chondrocytes apoptosis and CEP degeneration.** (**A**) CEP chondrocytes were isolated and treated with 100 μM TBHP with increasing concentrations (0, 0.1, 1, 10 μM) of VO-OHpic for 24 hours, CCK assay was conducted to evaluate the cell viability. (**B**) CEP chondrocytes were treated with increasing concentrations of TBHP and 1 μM VO-OHpic for 7 days and Alcian blue staining was conducted to examine the ECM production. (**C**) Flow cytometric analysis of endplate chondrocytes stained with Annexin V-FITC/PI. Percentage of apoptosis rates was expressed as means ± SD from three independent experiments. (**D**) CEP chondrocytes were pretreated with VO-OHpic (1 μM) for 18 hours, then 100 μM TBHP was added for 6 hours. Western blot was conducted to examine the protein levels of SOX9, COL2, MMP3, MMP13, BCL-2 and BAX. The band density of SOX9, COL2, MMP3, MMP13 and the ratio of BCL-2/BAX were quantified and normalized to control. (**E**) CEP chondrocytes were pretreated with VO-OHpic (1 μM) for 18 hours, then 100 μM TBHP was added for 6 hours. Immunofluorescence staining was conducted to examine the expression of COL2 (red). Scale bar = 20 μm. (**F**, **G**) Immunohistochemistry for COL2 and MMP3 in cartilage endplate from each group. Scale bar = 20 μm. The ratio of positive cells for COL2 and MMP3 was quantified under a microscope at 400× magnification using five sections from seven mice. Data are presented as mean ± SD from three independent experiments. ^*^P < 0.05, ^**^P < 0.01, ^***^P < 0.001, ^****^P < 0.0001.

Next, CEP cells were cultured with 100 μM TBHP supplemented with various concentrations of VO-OHpic. CCK8 assay was conducted to assess whether VO-OHpic administration could restore the cell viability. As shown in Figure 2A, VO-OHpic dose dependently resorted the cell viability and the most significant effect was found at 1 μM. Alcian blue staining showed that TBHP significantly inhibited CEP chondrocytes extracellular matrix production and this could be restored by 1 μM VO-OHpic co-treatment (Figure 2B). Western blot and immunofluorescence assay showed similar trend that VO-OHpic reversed the decline of chondrogenic markers, SOX9 and COL2, and the increase of matrix metalloproteinase, MMP3 and MMP13 (Figure 2D, 2E). Our results also demonstrated that VO-OHpic inhibited mitochondrial apoptosis induced by oxidative stress. The apoptotic rate of CEP chondrocytes was determined with Annexin V-FITC/PI Kit and we found that the average percentage of apoptotic cells in VO-OHpic group was significantly decreased compared with TBHP group (Figure 2C). In addition, VO-OHpic could reverse the elevated expression of BAX and decreased expression of Bcl-2 induced by TBHP treatment (Figure 2D). Our results demonstrated that VO-OHpic could inhibit oxidative stress induced mitochondrial apoptotic pathway. Moreover, our *in vivo* immunohistochemistry assay showed that VO-OHpic could reverse the decrease of type II collagen expression and the increase of MMP3 expression in the CEP of IDD model mice (Figure 2F, 2G). In summary, VO-OHpic could inhibit IDD pathological condition, including oxidative stress induced chondrocytes apoptosis and ECM degradation.

### VO-OHpic inhibited oxidative stress induced CEP calcification

**Figure 3:**
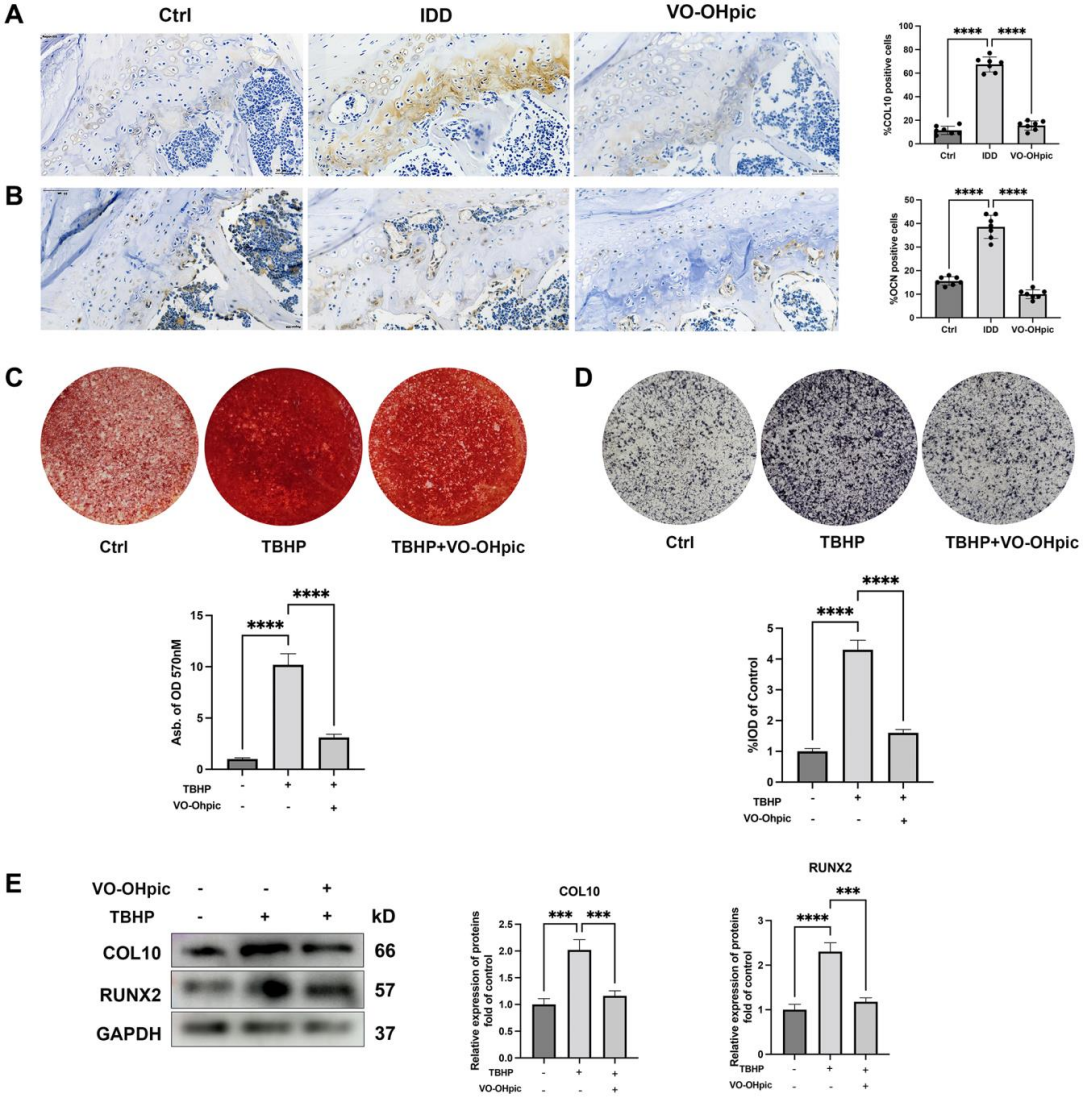
**VO-OHpic inhibited oxidative stress induced cartilage endplate calcification.** (**A**, **B**) Immunohistochemistry for COL10 and OCN in cartilage endplate from Ctrl group, IDD group and IDD+VO-OHpic group. Scale bar = 50 μm. The ratio of positive cells for COL10 and OCN were quantified under a microscope at 400× magnification using five sections from seven mice. (**C**) Alizarin Red staining for calcium deposition in endplate chondrocytes. Semi-quantitative analysis of the mineralized nodule in endplate chondrocytes. (**D**) ALP staining in endplate chondrocytes. Semi-quantitative analysis of alkaline phosphatase (ALP) activity in endplate chondrocytes. (**E**) CEP chondrocytes were treated with TBHP (100 μM) and VO-OHpic (1 μM) for 24 h and western blot was conducted to examine the protein levels of COL10 and RUNX2. The band density of COL10 and RUNX2 was quantified and normalized to control. Data are presented as mean ± SD from three independent experiments. ^***^P < 0.001, ^****^P < 0.0001.

Recent studies demonstrated that CEP degeneration and calcification could significantly decrease the oxygen and nutrient supply of intervertebral discs and is the leading cause to IDD [23]. Oxidative stress is an important detrimental stimulus for CEP chondrocytes hypertrophy and osteogenic differentiation [13]. To investigate whether VO-OHpic could inhibit endplate calcification, chondrocyte hypertrophic and osteogenic markers, OCN and COL10 were assessed using immunohistochemistry staining. As shown in Figure 3A, 3B, cartilage endplate of IDD mice model exhibited higher percentage of COL10 and OCN positive cells compared to the control group, while lower level of CON and COL10 positive cells were observed in cartilage endplate of mice in VO-OHpic treatment group. Also CEP chondrocytes were isolated and the effects of VO-OHpic in CEP chondrocytes osteogenic differentiation and mineralization induced by oxidative stress *in vitro* were investigated. As shown in Figure 3C, 3D, alizarin red staining and ALP activity analysis were conducted to examine calcium nodules. Consistent with previous studies, TBHP treatment significantly promoted CEP chondrocytes mineralized deposits formation and ALP activity, and this could be reversed by VO-OHpic co-treatment. Similar trends were observed during western blotting. VO-OHpic significantly inhibited oxidative stress induced chondrocytes hypertrophic and osteogenic markers COL10 and RUNX2 expression (Figure 3E).

### VO-OHpic protects CEP against degeneration and calcification via mitophagy stimulation

**Figure 4:**
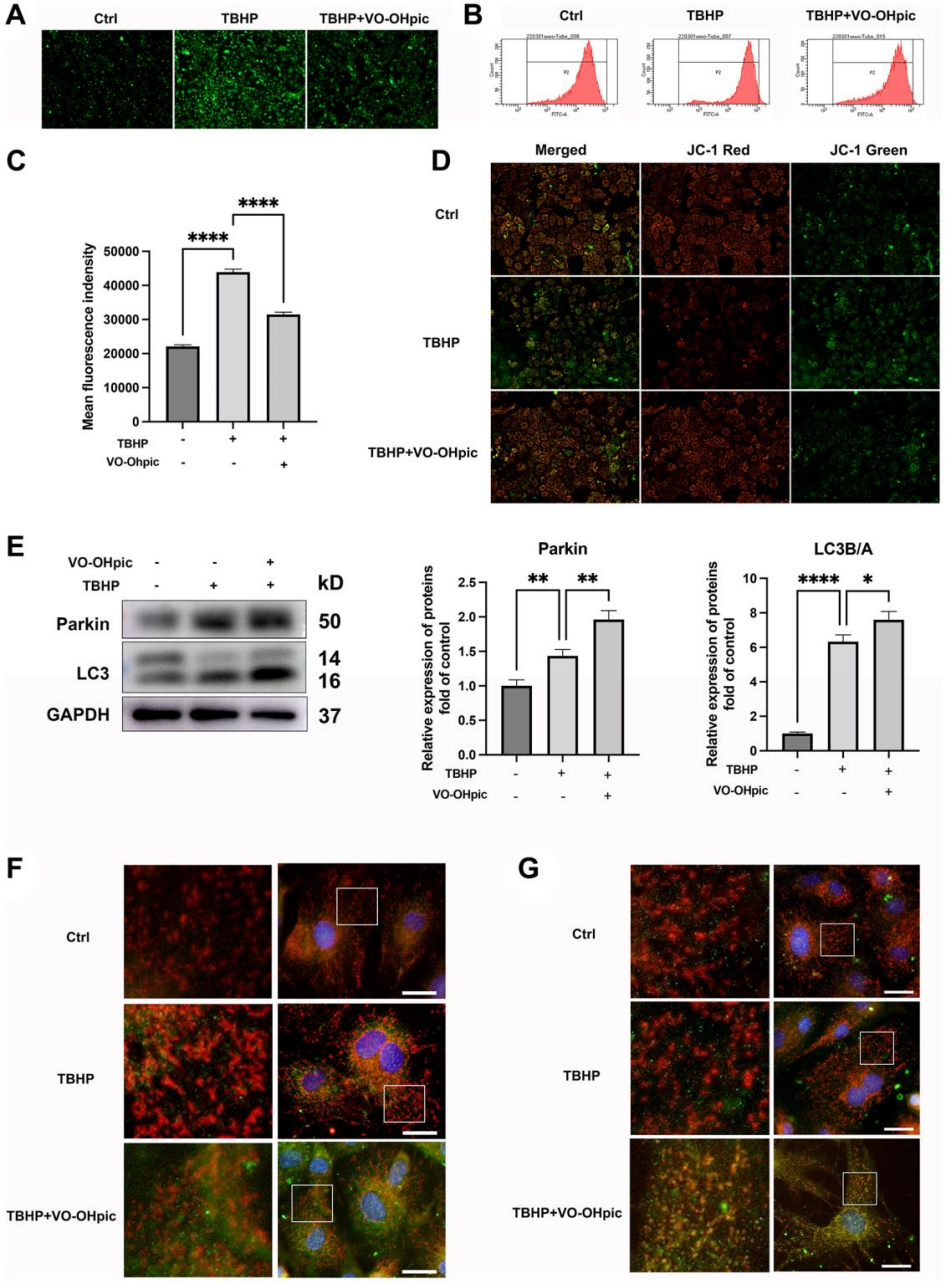
**VO-OHpic treatment promoted mitophagy in CEP chondrocytes.** (**A**) CEP chondrocytes were treated with TBHP (100 μM) and VO-OHpic (1 μM) for 24 h, representative fluorescence microscopy photomicrographs of intracellular ROS in chondrocytes. (**B**) ROS was detected by flow cytometric analysis after labeling with DCFH-DA. (**C**) The bar graphs show the mean fluorescence intensity of ROS levels in endplate chondrocytes. (**D**) Representative fluorescence microscopy photomicrographs of mitochondrial membrane potential (MMP) after incubating with JC-1. Red fluorescence was emitted by JC-1 aggregates in healthy mitochondria with polarized inner mitochondrial membranes, whereas green fluorescence was emitted by cytosolic JC-1 monomers, indicating MMP collapse. (**E**) CEP chondrocytes were pretreated with VO-OHpic (1 μM) for 18 hours, then 100 μM TBHP was added for 6 hours, western blot was conducted to examine the protein levels of parkin and LC3. The band density of parkin and LC3 was quantified and normalized to control. (**F**, **G**) Immunofluorescence staining was conducted to examine the expression and localization of LC3B, parkin (green) and mitochondria (red). Scale bar = 25 μm. Data are presented as mean ± SD from three independent experiments. ^*^P < 0.05, ^**^P < 0.01, ^****^P < 0.0001.

Cellular redox balance is indispensable to cartilage endplate homeostasis [24]. We next investigated whether VO-OHpic could inhibit TBHP induced excess ROS production and mitochondrial dysfunction. DCFH-DA analysis showed that VO-OHpic inhibited TBHP induced excess ROS production (Figure 4A). To quantify ROS production, flow cytometric analysis was conducted and VO-OHpic significantly inhibited ROS production which was induced by TBHP (Figure 4B, 4C). Next, mitochondrial dysfunction was assessed by alteration of mitochondrial membrane potential (MMP). An increased ratio of green JC-1 fluorescence compared to red JC-1 fluorescence was found in TBHP group, indicating TBHP reduced the mitochondrial membrane potential and led to mitochondrial dysfunction. However, VO-OHpic treatment restored the mitochondrial dysfunction and reversed the decrease of MMP with reduced ratio of green fluorescence to red fluorescence (Figure 4D).

Next, the role of mitophagy in the protective effect of VO-OHpic was investigated. Mitophagy related proteins were detected by western blot after TBHP with or without VO-OHpic treatment. As shown in Figure 4E, the LC3B/A ratio and parkin expression, which are regarded as indicators of mitophagy activation, were slightly increased after TBHP treatment, while VO-OHpic supplementation further promoted parkin expression and the LC3B/A ratio. Immunofluorescence staining was conducted to evaluate mitophagy process, and the yellow staining indicated the co-localization of autophagosome with mitochondria and mitophagy was activated. As shown in Figure 4F, 4G, our immunofluorescence staining analysis showed similar results that the autophagosomes were increased after VO-OHpic treatment with increased co-localization of LC3B and parkin with mitochondria.

**Figure 5:**
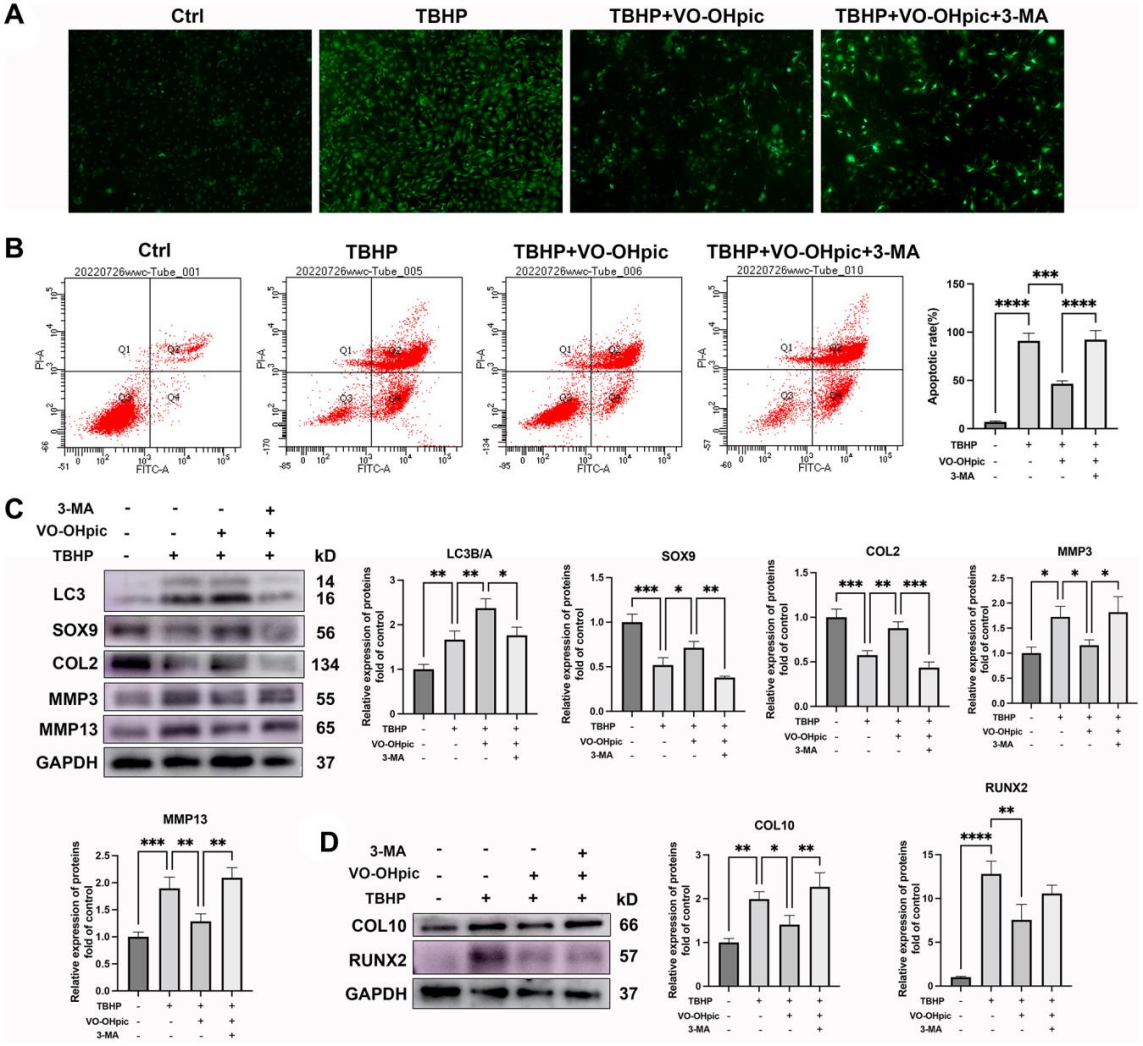
**VO-OHpic protects CEP against degeneration and calcification via mitophagy stimulation.** CEP chondrocytes were pretreated with 3-MA for 10 hours, then culture medium was changed with VO-OHpic (1 μM) for 18 hours and 100 μM TBHP was added for 6 hours. ROS production was evaluated with DCFH-DA staining, (**A**) Representative fluorescence microscopy photomicrographs of intracellular ROS in chondrocytes. (**B**) Flow cytometric analysis of endplate chondrocytes stained with Annexin V-FITC/PI. Percentage apoptosis rates were expressed as means ± SD. (**C**) Western blot was conducted to examine the protein levels of LC3, SOX9, COL2, MMP3 and MMP13. The band density of SOX9, COL2, MMP3, MMP13 and the ratio of BCL-2/BAX were quantified and normalized to control. (**D**) Western blot was conducted to examine the protein levels of CO10 and RUNX2. The band density of CO10 and RUNX2 was quantified and normalized to control. Data are presented as mean ± SD from three independent experiments. ^*^P < 0.05, ^**^P < 0.01, ^***^P < 0.001, ^****^P < 0.0001.

To investigate whether VO-OHpic protects CEP chondrocytes against degeneration via mitophagy, the autophagy inhibitor 3-MA was used in this study. As shown in Figure 5A, 3-MA partly abrogated the inhibitory effect of VO-OHpic in TBHP induced ROS overproduction. Annexin V/PI flowcytometry analysis showed that VO-OHpic inhibited TBHP induced CEP chondrocytes apoptosis, and this effect was inhibited by 3-MA co-treatment with increased apoptotic rate in CEP chondrocytes of 3-MA group (Figure 5B). The western blot results showed that VO-OHpic promoted the chondrogenic differentiation markers, SOX9 and COL2, inhibited ECM degrading enzymes and chondrocytes hypertrophic markers, MMP3, MMP13, COL10 and RUNX2 expression. However, in the 3-MA group, SOX9 and COL2 expression were down-regulated, MMP3, MMP13, COL10 and RUNX2 expression were up-regulated, indicating that the protective effect of VO-OHpic against CEP degeneration and calcification is through mitophagy stimulation (Figure 5C, 5D).

### Nrf-2 activation is required for VO-OHpic induced mitophagy process

**Figure 6:**
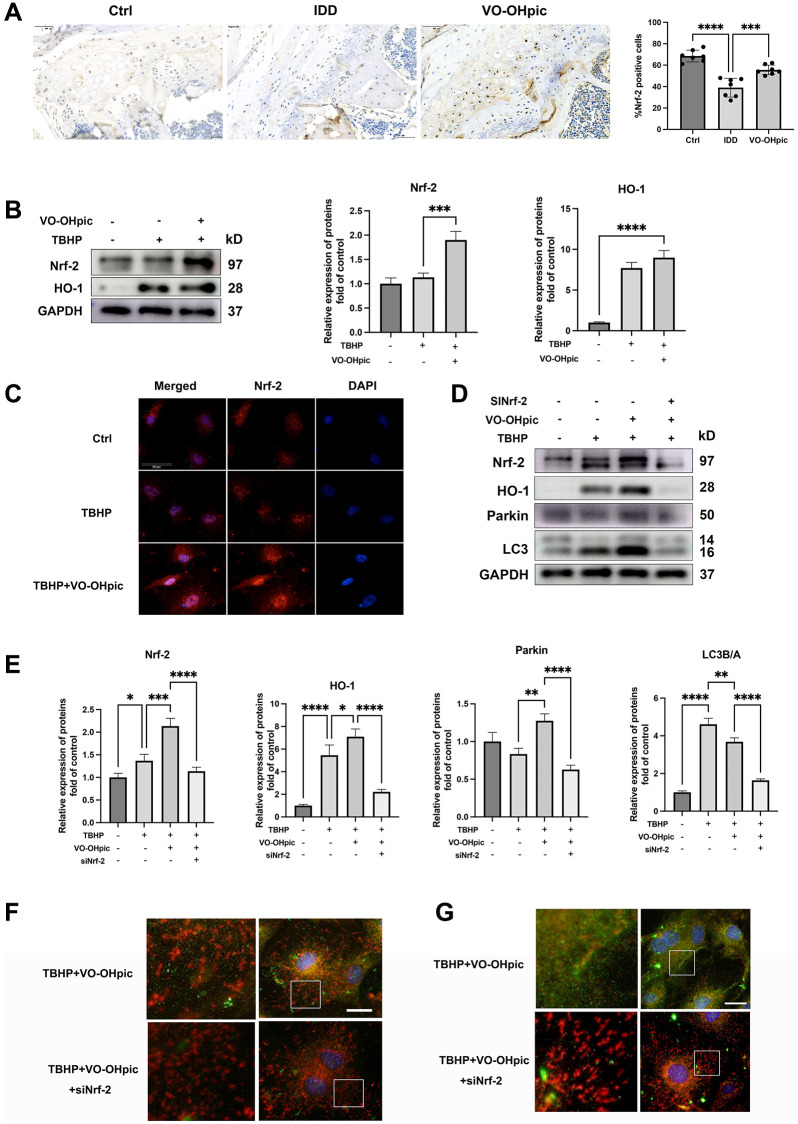
**Nrf-2 activation is required for VO-OHpic induced mitophagy process.** (**A**) Immunohistochemistry for Nrf-2 in cartilage endplate from Ctrl group, IDD group and IDD+VO-OHpic group. Scale bar = 50 μm. The ratio of positive cells for COL10 was quantified under a microscope at 400× magnification using five sections from seven mice. (**B**) CEP chondrocytes were pretreated with VO-OHpic (1 μM) for 18 hours, then 100 μM TBHP was added for 6 hours. Western blot was conducted to examine the protein levels of Nrf-2 and HO-1. The band density of Nrf-2 and HO-1 was quantified and normalized to control. (**C**) CEP chondrocytes were treated with TBHP (100 μM) and VO-OHpic (1 μM) for 24 h and immunofluorescence staining was conducted to examine the expression and localization of Nrf-2 (red). Scale bar = 20 μm. (**D**) Chondrocytes were transfected with Nrf-2 siRNA, and treated with TBHP (100 μM) and VO-OHpic (1 μM), western blot was conducted to examine the protein levels of Nrf-2, HO-1, parkin and LC3. (**E**) The band density of Nrf-2, HO-1, parkin and LC3 was quantified and normalized to control. (**F**, **G**) Chondrocytes were transfected with Nrf-2 siRNA, and treated with TBHP (100 μM) and VO-OHpic (1 μM). Immunofluorescence staining was conducted to examine the expression and localization of LC3B, parkin (green) and mitochondria (red). Scale bar = 25 μm. Data are presented as mean ± SD from three independent experiments. ^*^P < 0.05, ^**^P < 0.01, ^***^P < 0.001, ^****^P < 0.0001.

Nrf-2 was reported to be a major regulator of cellular redox balance and mitophagy [25]. There are reports demonstrating that Nrf-2 could directly regulate parkin expression, thus modulate cellular mitophagy process and exhibit anti-oxidant effect [19]. As shown in Figure 6A, immunohistochemistry assay of CEP showed that Nrf-2 was downregulated in the endplate of IDD mice, while VO-OHpic administration significantly promoted endplate chondrocytes Nrf-2 expression. To further investigate the role Nrf-2 in the protective effect of VO-OHpic, CEP chondrocytes were isolated and treated with 100 μM TBHP with or without VO-OHpic. Western blot analysis showed that VO-OHpic significantly promoted Nrf-2 and HO-1 proteins expression (Figure 6B). Immunofluorescence staining showed similar results that VO-OHpic significantly promoted red fluorescence labeled Nrf-2 nucleus translocation and expression (Figure 6C). Nrf-2 siRNA was then transfected into CEP chondrocytes and the role of Nrf-2/HO-1 pathway in the mitophagy process was investigated. As shown in Figure 6D, 6E, Nrf-2 knockdown inhibited VO-OHpic induced parkin upregulation and the elevated ratio of LC3II/I, immunofluorescence assay obtained similar trend that Nrf-2 knockdown inhibited VO-OHpic induced green fluorescence stained LC3B and parkin expression and co-localization with red fluorescence stained mitochondria (Figure 6F, 6G). These results indicated that VO-OHpic induced mitophagy process was partly Nrf-2 dependent.

### VO-OHpic inhibited oxidative stress induced CEP degeneration and chondrocytes ferroptosis via activating Nrf-2

**Figure 7:**
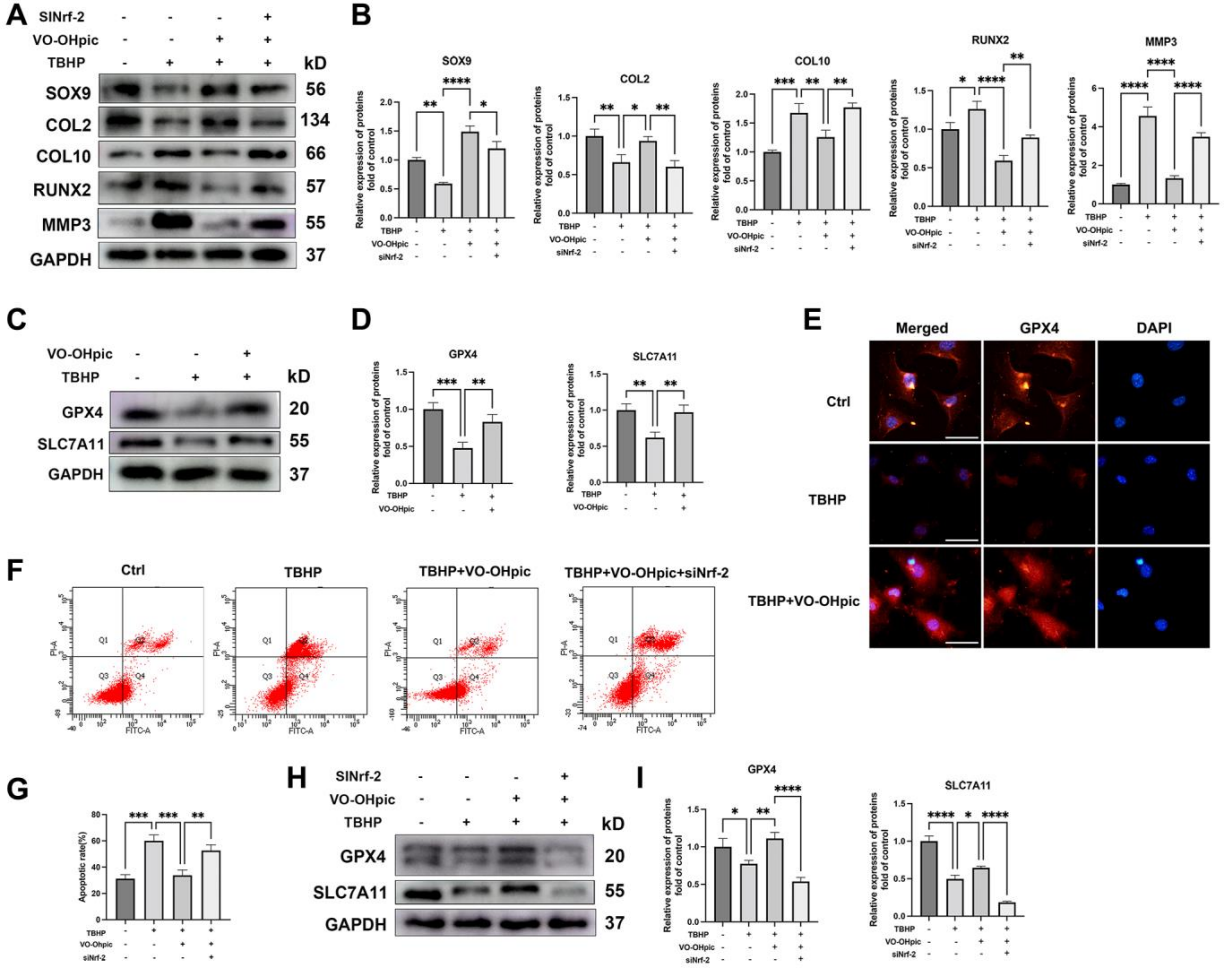
**VO-OHpic inhibited oxidative stress induced CEP degeneration and chondrocytes ferroptosis via activating Nrf-2.** Chondrocytes were transfected with Nrf-2 siRNA, and treated with TBHP (100 μM) and VO-OHpic (1 μM). (**A**) Western blot was conducted to examine the protein levels of SOX9, COL2, COL10, RUNX2 and MMP3. (**B**) The band density of Nrf-2, HO-1, parkin and LC3 was quantified and normalized to control. (**C**) CEP chondrocytes were treated with TBHP (100 μM) and VO-OHpic (1 μM) for 24 h and western blot was conducted to examine the protein levels of GPX4 and SLC7A11. (**D**) The band density of GPX4 and SLC7A11 was quantified and normalized to control. (**E**) Immunofluorescence staining was conducted to examine the expression and localization of GPX4 (red). (**F**, **G**) Flow cytometric analysis of endplate chondrocytes stained with Annexin V-FITC/PI. Percentage apoptosis rates were expressed as means ± SD. (**H**) Chondrocytes were transfected with Nrf-2 siRNA, and treated with TBHP (100 μM) and VO-OHpic (1 μM), western blot was conducted to examine the protein levels of GPX4 and SLC7A11. (**I**) The band density of GPX4 and SLC7A11 was quantified and normalized to control. Data are presented as mean ± SD from three independent experiments. Scale bar = 20μm. ^*^P < 0.05, ^**^P < 0.01, ^***^P < 0.001, ^****^P < 0.0001.

Next, Nrf-2 siRNA was transfected into CEP chondrocytes and the role of Nrf-2/HO-1 in TBHP induced CEP degeneration and apoptosis was investigated. As shown in Figure 7A, 7B, Nrf-2 siRNA transfection abrogated the protective effect of VO-OHpic and promoted MMP3, COL10 and RUNX2 expression, and inhibited SOX9 and COL2 expression. These results demonstrated the essential role of Nrf-2 in VO-OHpic ameliorating cartilage endplate degeneration. Then, chondrocyte apoptotic rates were assessed by Annexin V- FITC/PI flowcytometric analysis. VO-OHpic inhibited chondrocyte apoptosis compared with the TBHP group, while Nrf-2 inhibition abrogated the protective effect of VO-OHpic in cell viability (Figure 7F, 7G). Growing evidences have reported the important role of ferroptosis in IDD, however, the connection between ferroptosis and CEP degeneration still needs to be further explored. Nrf-2 has been demonstrated to be a key regulator of ferroptosis. We next investigated the role of ferroptosis in the CEP degeneration and its regulation. As shown in Figure 7C–7E, immunofluorescence analysis and western blot results showed that TBHP significantly decreased proteins expression of GPX4 and SLC7A11, while VO-OHpic treatment reversed the decreased protein levels of GPX4 and SLC7A11 in endplate chondrocytes, which indicated that VO-OHpic inhibited oxidative stress induced endplate chondrocyte ferroptosis. Nrf-2 knockdown blocked the VO-OHpic mediated up-regulation of SLC7A11 and GPX4, indicating that VO-OHpic decreased chondrocytes ferroptosis via Nrf-2 activation (Figure 7H, 7I). In conclusion, our results indicated that VO-OHpic inhibited oxidative stress induced CEP degeneration and chondrocytes ferroptosis via activating Nrf-2.

## DISCUSSION

Intervertebral disc degeneration is the pathological foundation of a series spinal diseases, including lumbar disc herniation and low back pain. Cartilage endplate locates at both ends of the intervertebral disc and is responsible for anchoring the vertebral body. CEP degeneration and calcification has recently been demonstrated to be an important contributor to IDD by decreasing the nutrition and oxygen supply to the NP and AF [4]. However, previous studies mainly focused on the degeneration of NP and AF, the mechanisms underlying cartilage endplate degeneration has not been fully elucidated. Accordingly, targeting cartilage endplate degeneration to improve nutrient and oxygen supply of the intervertebral disc is drawing increasing interest. Recent study reported that PTEN was upregulated in degenerative nucleus pulposus and was involved in IDD process [8]. Lin Y et al. found that PTEN inhibitor VO-OHpic could inhibit proinflammatory cytokines and oxidative stress induced NP degeneration. However, their results lack *in vivo* experiments to demonstrate the effect of VO-OHpic in protecting against IDD. Moreover, the role of PTEN in CEP degeneration and calcification remains elusive [9]. VO-OHpic has been demonstrated to be an efficient PTEN inhibitor and effective in many diseases. Moreover, previous studies have demonstrated that VO-OHpic is safe *in vivo* and the inhibition effect of PTEN is reversible, growing interests have been focused on the clinical translational potential of VO-OHpic [16]. In the present study, our experiments demonstrated that VO-OHpic ameliorated CEP degeneration and IDD development. VO-OHpic could inhibit CEP chondrocytes ferroptosis and promote mitophagy process via activating Nrf-2/HO-1 pathway, which subsequently inhibited CEP chondrocytes apoptosis, osteogenic differentiation and ECM degrading enzymes expression.

Previous clinical evidences have demonstrated that intervertebral disc degeneration was positively correlated to cartilage endplate degeneration. The incidence of lumbar disc herniation and low back pain is much higher in patients with endplate osteochondritis [26]. Inhibiting CEP calcification and degeneration could improve the nutrient supply status of intervertebral discs and inhibit the IDD process [3, 27]. In the present study, IDD mice model was established and we first investigated whether VO-OHpic could protect against IDD process *in vivo*. The IDD mice model used in this study was established by transection of bilateral facet joints which could cause intervertebral disc instability and CEP degeneration [28]. As expected, IDD mice model exhibited obvious intervertebral disc degeneration with reduced NP and increased calcified CEP with obvious bony tissues and chondrocyte lacuna, while VO-OHpic administration partly reversed the detrimental effect of VO-OHpic with increased amounts of ECM in nucleus pulposus and cartilage endplate. These results demonstrated that VO-OHpic could inhibit IDD development and ameliorate CEP calcification, thus providing a promising treatment strategy for IDD.

Various risk factors related to IDD, including mechanical overload, instability, and trauma could lead to chondrocytes mitochondrial dysfunction, and thus promote excess ROS production and decrease in the cell viability [1, 29]. Oxidative stress has been demonstrated to be a common pathological factor for apoptosis and calcification in cells, including chondrocytes [30]. Our results showed that VO-OHpic significantly promoted endplate chondrocytes chondrogenic differentiation genes, SOX9 and COL2 expression, and inhibited ECM degrading enzymes, MMP3 and MMP13 expression, thus promoting formation of cartilage matrix. Oxidative stress could lead to mitochondrial dysfunction and activate mitochondrial apoptosis pathway with increasing apoptosis-related proteins, such as Bax, and decreasing anti-apoptosis-related proteins, such as Bcl-2. Our results showed that VO-OHpic promoted Bcl-2 expression while inhibited Bax expression, suggesting that VO-OHpic may protect CEP chondrocytes against oxidative stress induced apoptosis. ALP activity assay and Alizarin red staining, which are reliable osteogenic markers, showed that VO-OHpic could inhibit oxidative stress induced chondrocytes calcification. CEP chondrocytes osteogenic markers, RUNX2 and COL10 were down-regulated after VO-OHpic treatment. Together, our *in vitro* results demonstrated that VO-OHpic could inhibit oxidative stress induced CEP chondrocytes apoptosis and osteogenic differentiation, thus inhibit CEP calcification and degeneration.

Mitophagy is a newly identified mitochondrial autophagy process which could clear damaged mitochondria and inhibit excess ROS production [15]. The mitophagy marker, parkin was found decreased in NP cells during IDD development, activation of mitophagy could restore mitochondrial homeostasis and inhibit IDD development [31]. Recently, Yao et al. reported that VO-OHpic could protect endothelial progenitor cells against oxidative stress induced mitochondrial dysfunction and maintain redox balance [32], our results also demonstrated that VO-OHpic could inhibit oxidative stress induced mitochondrial apoptosis and CEP degeneration, however, how VO-OHpic modulates CEP chondrocytes redox balance and ameliorates IDD remain unclear. Therefore, we were curious whether VO-OHpic could regulate CEP chondrocytes mitophagy and its underlying mechanisms. We first demonstrated that VO-OHpic could inhibit TBHP induced excess ROS production and mitochondrial dysfunction, also our immunofluorescence staining showed that microtubule-associated proteins 1B light chain 3B (LC3B) and parkin were recruited to the outer membrane of mitochondria after continuous VO-OHpic stimulation, which suggested that chondrocyte mitophagy was activated.

To demonstrate the role of mitophagy in the protective effect of VO-OHpic in apoptosis or calcification of CEP chondrocytes, the classic inhibitor of autophagolysosome formation 3-MA was used in this study. VO-OHpic induced mitophagy was inhibited with 3-MA treatment, which was indicated by the decreased LC3B/A ratio. Meanwhile, 3-MA treatment attenuated the protective effects of VO-OHpic on CEP chondrocytes with increased incidence of apoptosis and cartilage matrix degrading enzymes. Additionally, the increased osteogenic markers and decreased chondrogenic markers indicated 3-MA reversed the beneficial effect of VO-OHpic in CEP chondrocytes degeneration and calcification. Thus, VO-OHpic protected chondrocytes against apoptosis and calcification via mitophagy.

When Nrf-2 is free from the interaction with Keap1, it translocates into the nucleus and activates the cytoprotection mechanism against environmental stimuli and oxidative stress [17]. Studies have shown that Nrf-2 expression was decreased with IDD development and Nrf-2 targeting activation has been proved to be a promising target for preventing IDD [19]. Several studies also demonstrated that Nrf-2 could directly regulate parkin protein expression and promote mitophagy process [31]. Our results showed that Nrf-2 protein was activated with VO-OHpic treatment, Nrf-2 knockdown not only partly inhibited the protective effect of VO-OHpic, but also inhibited the mitophagy process, suggesting that Nrf-2 plays important roles in the protective effect of VO-OHpic in CEP chondrocytes.

CEP chondrocytes apoptosis would reduce the ECM production and the CEP self-renewal ability, which is the direct cause of CEP degeneration [26]. Ferroptosis has been reported to be involved in many diseases, such as osteoporosis, Parkinson’s syndrome, and tumor genesis [31]. It has been reported recently that IDD risk factors, including oxidative stress and inflammation could promote chondrocytes ferroptosis and decrease the cell viability [32]. However, the participation of ferroptosis in CEP remains unclear. Besides regulating redox balance and inhibiting mitochondrial dysfunction, there are also studies reporting that Nrf-2 could regulate the key ferroptosis marker, GPX4 and FTL/FTH1 [17]. Therefore, we also investigated whether ferroptosis was involved in IDD development and whether VO-OHpic inhibited ferroptosis via activating Nrf-2/HO-1. Our results demonstrated that ferroptosis was activated under TBHP stimulation with decreased ferroptosis related proteins, GPX4 and SLC7A11 expression, and VO-OHpic co-treatment partly inhibited THBP induced ferroptosis with upregulated GPX4 and SLC7A11 expression. Moreover, Nrf-2 knockdown partly abrogated the protective effect of VO-OHpic in chondrocytes ferroptosis. Our results suggest that ferroptosis takes parts in the CEP degeneration and VO-OHpic could inhibit ferroptosis via activating Nrf-2/HO-1 pathway.

In conclusion, our study provides evidence that VO-OHpic might have anti-apoptosis, anti-degeneration and anti-calcification effects in cartilage endplates, and the underlying mechanism of action may be related to Nrf-2/HO-1 mediated ferroptosis inhibition and mitophagy activation, thus alleviated redox imbalance and mitochondrial dysfunction and eventually improved cell survival. Our results suggest that VO-OHpic could be an alternative effective medicine for IDD treatment.

## MATERIALS AND METHODS

### Reagents

VO-OHpic (S8174) and 3-Methyladenine (3-MA) (S2762) were purchased from Selleck (USA). Tert-butyl hydroperoxide (TBHP) was obtained from Sigma-Aldrich (St. Louis, MO, USA).

### Cell isolation and culture

CEP chondrocytes were isolated from the CEP of 7-day-old C57/BL6 male mice according to our previous study [33]. Briefly, cartilage endplate was dissected and the surrounding muscle and ligament tissue were carefully removed in the dissecting microscope. After digesting with 0.25% EDTA trypsin for 30 min and Type II collagenase for 1 h at 37°C, cartilage endplate was minced into 1 mm^3^ in size. Following another 5 hours of digestion with the collagenase and washed with PBS for three times, the primary CEP chondrocytes were collected and cultured in DMEM/F12 medium containing 10% FBS and 100 mg/mL streptomycin sulfate as well as 100 U/mL penicillin at 37°C. The first or second passage of chondrocytes were chosen to use in the present study.

### Alcian blue and alizarin red staining

To determine the CEP chondrocytes ECM production, Alcian blue staining was conducted. The CEP chondrocytes were first seeded in 12-well plate at density of 1 × 10^6^/ml with a volume of 100 ul, and after adherent for about 30 min, the chondrocytes were then washed twice with PBS and cultured with chondrogenic differentiation medium (Cyagen Biosciences, Guangzhou, China) 2 ml/well for 7 days. After washing with PBS and fixed with 4% paraformaldehyde for 15 min, chondrocytes were incubated with Alcian Blue solution for 30 min. Then chondrocytes were washed by distilled water for three times, and images were captured with an inverted microscope (Nikon, Tokyo, Japan).

The formation of mineralized nodules *in vitro* is a manifestation of the osteoblastic ability of chondrocytes, which can be analysed by alizarin red staining. Briefly, CEP chondrocytes were seeded in 24-well plates at a density of 1×10^5^ cells per well. Osteogenic differentiation culture medium (Cyagen Biosciences, Guangzhou, China) was added in chondrocytes when the cell density reached 80% confluence. Osteogenic differentiation induction time lasts for 3 weeks. After being washed with distilled water and fixed with 4% paraformaldehyde, the cells were stained with alizarin red solution (Cyagen Biosciences, Guangzhou, China) for 30 min at room temperature. The number of mineralized nodules is quantitatively assessed by spectrophotometry. More specially, after dissolving using 10% (wt/vol) cetylpyridinium chloride (Sigma-Aldrich, St. Louis, MO, USA) for 1 h, the dye was quantified via spectrophotometric absorbance measurements of optical density at 570 nm.

### Alkaline phosphatase staining and ALP activity assay

To further assess the degree of chondrocyte mineralization, ALP staining was performed in addition to alizarin red staining. In general, the activity of ALP peaked after 7 days of osteogenic induction culture, therefore, cells were stained with ALP staining kit according to the manufacturer’s instructions (P0321S, Beyotime, China) on the seventh day of induction. Briefly, chromogenic substrate solution and standard working solution were prepared using p-nitrophenyl phosphate (pNPP) substrate, then 50 ml of cell lysates and 50 ml of appropriate buffer solution were mixed and incubated for 10 min at 37°C before stopping buffer was added. BCA protein assay kit (Boster, China, AR0146) was used to determine the total protein concentration. ALP activity was determined as the OD value at 405 nm per milligram of total protein.

### Cell viability assay

CCK-8 assay was conducted to assess the viability of CEP chondrocytes. The first or second passage of CEP chondrocytes were seeded into 96-well plates at a density of 3 × 10^3^ cells/well with five replicate wells for 24 h. After adhesion and treatment, 10 μL of CCK-8 test solution was added to each well. The cells were incubated for 1 h, and the absorbance at 450 nm was obtained by a microplate reader. The percentage of cell viability was normalized according to the viability of untreated cells.

### Annexin V-FITC/PI staining and flow cytometry

Annexin V-FITC/PI staining and flow cytometry were conducted to examine the apoptotic effect of CEP chondrocytes. After treatment, the cells were collected by centrifugation and stained with Annexin V-FITC/PI apoptosis detection kit (MA0220, Meilunbio, Dalian, China) for 20 min in the darkness after the last centrifugation. The sum of early apoptotic cells (annexin V+/PI−) and late apoptotic cells (annexin V+/PI+) divided by normal cells is the CEP chondrocyte apoptosis rate.

### Assessment of intracellular ROS and mitochondrial membrane potential (MMP)

Excessive accumulation of ROS is the main cause of oxidative stress, which plays an important role in IDD. So, the intracellular ROS production was assessed using a Reactive Oxygen Species Assay Kit (S0033, Beyotime, Shanghai, China) following the manufacturer’s guidelines. The CEP cells were washed three times with serum-free DMEM/F12 before being treated with TBHP or VO-OHpic. Then DCFH-DA was diluted to 10 uM and added into cells for 30 min in the dark. After washing with serum-free DMEM/F12, the mean fluorescence intensity was calculated using a FACSCalibur flow cytometer, (BD Biosciences, Franklin Lakes, NJ, USA).

MMP decreased in the early stage of apoptosis, the MMP changes were evaluated via mitochondrial membrane potential kit (C2006, Beyotime, Shanghai, China). After washing three times with PBS, the cells were then incubated with the JC-1 staining working solution and equal volume of serum-free medium for 30 min at 37°C in the absence of light. After washing with ice-cold JC-1 washing buffer for 2 times, the cells were analysed using the fluorescence microscope (Axio Observer 3; Carl Zeiss).

### Western blotting analysis

CEP chondrocytes were seeded at a density of 2 × 10^5^ cells/well and treatment was given when the cell density reached 80% confluence. After intervention, cells in each well were washed with PBS three times and added with 100 ul RIPA lysis buffer supplemented with 1% proteinase inhibitor cocktail and 1% phosphatase inhibitor cocktail. Then lysates were collected and lysed for 30 min on ice. After determination of protein concentration, 25 ug protein sample was separated by 10% SDS-PAGE gel and electrotransferred to methanol-activated PVDF membrane. After blocking with 5% non-fat dry milk for 1 hour, PVDF membranes were incubated with targeted primary antibodies overnight at 4°C. After three times of washing with TBST and 1 hour of incubation with corresponding peroxidase-conjugated secondary antibodies at room temperature, bands were detected using the enhanced chemiluminescence reagents (Thermo Fisher) and analyzed with BandScan scanner (Bio-Rad, Hercules, CA, USA). Band density was quantified using image J version 1.48 and normalized using GAPDH. The primary antibodies were as follows: Type II collagen (#28459-1-AP, Proteintech), SOX-9 (#A00177-2, CST), MMP3 (#17873-1-AP, Proteintech), MMP13 (#18165-1-AP, Proteintech), OCN (#23418-1-AP, Proteintech), COL10A1 (#BA 2023, Boster), RUNX2 (#PB0171, Boster), SLC7A11 (#26864-1-AP, Proteintech), GPX4 (#67763-1-Ig, Proteintech), PARKIN (#14060-1-AP, Proteintech), Bcl-2 (#26593-1-AP, Proteintech), BAX (#60267-1-Ig, Proteintech), Nrf2 (#16396-1-AP, Proteintech), HO-1 (#BM4010, Boster), LC3A/B (#4108, CST), GAPDH (#10494-1-AP, Proteintech).

### Immunofluorescence staining

CEP chondrocytes were seeded in 24-well plates and subjected to different treatment when reached the appropriate cell density. After fixation and permeabilization, the cells were blocked with 5% BSA for 1 h at 37°C. The cells need washing thrice with PBS before fixation, permeabilization and blocking procedure. Subsequently, the cells were incubated with the primary antibodies against GPX4 (1:500), COL2 (1:500) and Nrf2(1:200) at 4°C overnight and then were treated with Cy3-conjugated goat anti-rabbit secondary antibody (#A0516, Beyotime, Shanghai, China 1:500) in the darkness for 1 h at 37°C. And the 4,6-diamidino-2-phenylindole (DAPI) treatment for 10 min is the last step. Accordingly, three times of washing with PBS before incubation with primary and secondary antibody is an unavoidable part of staining. Fluorescence microscopy (Axio Observer 3; Carl Zeiss) was used to determine the fluorescence expression variation of corresponding protein.

The fluorescence examination of the mitophagy process was conducted by the colocalization of mitochondria and autophagy indices. After treatment, the cells were washed three times with serum-free DMEM/F12 before incubating with diluted Mito-Tracker Red CMXRos solution (#C1049B, Beyotime, Shanghai, China 1:500) in the dark at 37°C for 30 min. The next series of steps are 20 min of fixation with 4% paraformaldehyde at room temperature, 5 min of membrane penetration with 0.1% Triton X-100 and 1 h of blocking with 5% BSA at 37°C. Following incubation with primary antibodies against Parkin (1:200), LC3B (1:200) at 4°C overnight, the cells were treated with FITC-conjugated goat anti-rabbit secondary antibody (A0562, Beyotime, Shanghai, China 1:500) in the dark at 37°C for 1.5 h. At last, the cells were stained with 4,6-diamidino-2-phenylindole (DAPI) for 10 min. Before each step, rinsing cells with PBS for 3 times, 5 min each time, was indispensable. Fluorescence microscopy (Axio Observer 3; Carl Zeiss) was used to capture the images and detect the fluorescence expression difference of corresponding protein.

### siRNA transfection

CEP chondrocytes were firstly seeded at the density of 1 × 10^5^ cells/ml in 6-well plates, and in principle, siRNA can be added when cells were cultured for more than 24 h after adherence. Specifically, following removal of the primary cell culture medium, siNrf-2 was then added into cells using riboFECTTMCP kit, a transfection complex containing riboFECTTMCP Buffer, riboFECTTMCP Reagent and self-prepared P/S-free DMEM/F12, when cell density reached 40% confluence. The volume of transfection system was 2 ml/well and the final concentration of siNRF2 was 100 nM. The medium was replaced 24–72 h after transfection, and the specific transfection time was determined by the time when the cell density reached 80% confluence. After transfection, the total protein was extracted and western blot analysis was conducted to assess the efficiency of endogenous Nrf-2 protein knockdown. The most effective siNrf-2 was screened for subsequent study. SiRNA targeting Nrf-2 mRNA and siControl were synthesized and purchased from Ribo Bio (Ribobio Co. Ltd., Guangzhou., China).

### Animal grouping and treatment

Twenty-one eight weeks old male C57BL/6 mice were randomly divided into 3 groups: control group, IDD group and IDD+VO-OHpic group. The IDD model, established by surgically-induced instability, had previously been published [34]. Briefly, the L4/5 bilateral facet joints, supra- and interspinous ligaments were transected surgically by microscissors using a surgical microscope to induce supra-physiologic movement. Mice in the IDD+VO-OHpic group were treated with intraperitoneal injection of VO-OHpic (10 mg/kg), dissolved in 60 ul vehicle (10% DMSO, 40% PEG300, 5% Tween-80, and 45% saline) every other day for 12 weeks. Mice in the control group were injected intraperitoneally with 60 ul vehicle, without containing VO-OHpic (10% DMSO, 40% PEG300, 5% Tween-80, and 45% saline). Mice were euthanized after 12 weeks treatment and corresponding spinal tissue were collected for the following micro-CT, histology, and immunohistochemistry analysis. All animal experiments were approved by the Animal Care Committee of Shandong Provincial Hospital Shandong University.

### Micro-CT

After fixation in 4% paraformaldehyde for 24–48 hours, the muscles and ligaments around the lumbar spine were dissected. Next, the microarchitecture of the surgical modeling segment was scanned using micro-computed tomography (uCT, Scanco Viva-CT80, Scanco Medical AG, Basserdorf, Switzerland), to evaluate the calcification of upper and lower cartilage endplates of the L4/5 intervertebral disc, with the resolution of 13.0 μm, 55 kVp, and 145 μA. With data processing and 3D reconstruction software, direct 3D measuring techniques were used to calculate the following parameters: intervertebral disc height and bone volume/tissue volume (BV/TV).

### Immunohistochemistry

Following micro-CT examination, the lumbar segments were decalcified in 10% EDTA solution for 1 month, then embedded in paraffin wax and sectioned at 4 μm thickness in mid-sagittal plane. To measure and quantify the severity of IDD, we performed H&E staining and histological analyses. The degree of IDD was evaluated separately by three persons blind to this study. For immunohistochemistry, after deparaffinization with xylene and blocking with PBS containing 5% BSA for 30 minutes at 37°C, the sections were then incubated with primary antibodies to COL2, MMP3, COL10, OCN, and Nrf2 overnight at 4°C, and then biotinylated goat anti-rabbit secondary antibodies were incubated for 30 min. The sections were then incubated for 10 min with DAB and counterstained with hematoxylin.

### Statistical analysis

One-way ANOVA followed by Tukey’s test was used for multiple comparisons in WB and immunohistochemistry analyses. A one-way ANOVA with Dunnett’s test and a Student’s *t*-test were used for comparison in WB data expressed as relative fold change. *P* < 0.05 was considered to be statistically significant. All analyses were performed with GraphPad Prism software (Version 9.0).

### Data availability statement

All data of the current study are available from the corresponding author on reasonable request.

## References

[1] Zhang YG, Sun Z, Zhang Z, Liu J, Guo X. Risk factors for lumbar intervertebral disc herniation in Chinese population: a case-control study. Spine (Phila Pa 1976). 2009; 34:E918–22. 10.1097/BRS.0b013e3181a3c2de 19940721

[2] Daly C, Ghosh P, Jenkin G, Oehme D, Goldschlager T. A Review of Animal Models of Intervertebral Disc Degeneration: Pathophysiology, Regeneration, and Translation to the Clinic. Biomed Res Int. 2016; 2016:5952165. 10.1155/2016/5952165 27314030 PMC4893450

[3] Ding Y, Jiang J, Zhou J, Wu X, Huang Z, Chen J, Zhu Q. The effects of osteoporosis and disc degeneration on vertebral cartilage endplate lesions in rats. Eur Spine J. 2014; 23:1848–55. 10.1007/s00586-014-3324-9 24806259

[4] Ariga K, Miyamoto S, Nakase T, Okuda S, Meng W, Yonenobu K, Yoshikawa H. The relationship between apoptosis of endplate chondrocytes and aging and degeneration of the intervertebral disc. Spine (Phila Pa 1976). 2001; 26:2414–20. 10.1097/00007632-200111150-00004 11707702

[5] Li J, Yen C, Liaw D, Podsypanina K, Bose S, Wang SI, Puc J, Miliaresis C, Rodgers L, McCombie R, Bigner SH, Giovanella BC, Ittmann M, et al. PTEN, a putative protein tyrosine phosphatase gene mutated in human brain, breast, and prostate cancer. Science. 1997; 275:1943–7. 10.1126/science.275.5308.1943 9072974

[6] Georgescu MM. PTEN Tumor Suppressor Network in PI3K-Akt Pathway Control. Genes Cancer. 2010; 1:1170–7. 10.1177/1947601911407325 21779440 PMC3092286

[7] Pulido R. PTEN Inhibition in Human Disease Therapy. Molecules. 2018; 23:285. 10.3390/molecules23020285 29385737 PMC6017825

[8] Xi Y, Ma J, Chen Y. PTEN promotes intervertebral disc degeneration by regulating nucleus pulposus cell behaviors. Cell Biol Int. 2020; 44:583–92. 10.1002/cbin.11258 31663655

[9] Lin Y, Guo W, Chen KW, Xiao ZM. VO-OHpic attenuates intervertebral disc degeneration via PTEN/Akt pathway. Eur Rev Med Pharmacol Sci. 2020; 24:2811–9. 10.26355/eurrev_202003_20642 32271398

[10] Sun K, Luo J, Guo J, Yao X, Jing X, Guo F. The PI3K/AKT/mTOR signaling pathway in osteoarthritis: a narrative review. Osteoarthritis Cartilage. 2020; 28:400–9. 10.1016/j.joca.2020.02.027 32081707

[11] Mak LH, Vilar R, Woscholski R. Characterisation of the PTEN inhibitor VO-OHpic. J Chem Biol. 2010; 3:157–63. 10.1007/s12154-010-0041-7 21643420 PMC2957887

[12] Johnson TA, Singla DK. PTEN inhibitor VO-OHpic attenuates inflammatory M1 macrophages and cardiac remodeling in doxorubicin-induced cardiomyopathy. Am J Physiol Heart Circ Physiol. 2018; 315:H1236–49. 10.1152/ajpheart.00121.2018 30095997 PMC6297808

[13] Yang RZ, Xu WN, Zheng HL, Zheng XF, Li B, Jiang LS, Jiang SD. Involvement of oxidative stress-induced annulus fibrosus cell and nucleus pulposus cell ferroptosis in intervertebral disc degeneration pathogenesis. J Cell Physiol. 2021; 236:2725–39. 10.1002/jcp.30039 32892384 PMC7891651

[14] Wu H, Chen Q. Hypoxia activation of mitophagy and its role in disease pathogenesis. Antioxid Redox Signal. 2015; 22:1032–46. 10.1089/ars.2014.6204 25526784

[15] Zhang Z, Xu T, Chen J, Shao Z, Wang K, Yan Y, Wu C, Lin J, Wang H, Gao W, Zhang X, Wang X. Parkin-mediated mitophagy as a potential therapeutic target for intervertebral disc degeneration. Cell Death Dis. 2018; 9:980. 10.1038/s41419-018-1024-9 30250268 PMC6155159

[16] Bin J, Bai T, Zhao Q, Duan X, Deng S, Xu Y. Parkin overexpression reduces inflammation-mediated cardiomyocyte apoptosis through activating Nrf2/ARE signaling pathway. J Recept Signal Transduct Res. 2021; 41:451–6. 10.1080/10799893.2020.1825488 33012239

[17] Zhang X, Yu Y, Lei H, Cai Y, Shen J, Zhu P, He Q, Zhao M. The Nrf-2/HO-1 Signaling Axis: A Ray of Hope in Cardiovascular Diseases. Cardiol Res Pract. 2020; 2020:5695723. 10.1155/2020/5695723 32411446 PMC7204387

[18] Lepetsos P, Papavassiliou AG. ROS/oxidative stress signaling in osteoarthritis. Biochim Biophys Acta. 2016; 1862:576–91. 10.1016/j.bbadis.2016.01.003 26769361

[19] Tang Z, Hu B, Zang F, Wang J, Zhang X, Chen H. Nrf2 drives oxidative stress-induced autophagy in nucleus pulposus cells via a Keap1/Nrf2/p62 feedback loop to protect intervertebral disc from degeneration. Cell Death Dis. 2019; 10:510. 10.1038/s41419-019-1701-3 31263165 PMC6602960

[20] Wu J, Xue R, Wu M, Yin X, Xie B, Meng Q. Nrf2-Mediated Ferroptosis Inhibition Exerts a Protective Effect on Acute-on-Chronic Liver Failure. Oxid Med Cell Longev. 2022; 2022:4505513. 10.1155/2022/4505513 35480867 PMC9036161

[21] Martin D, Rojo AI, Salinas M, Diaz R, Gallardo G, Alam J, De Galarreta CM, Cuadrado A. Regulation of heme oxygenase-1 expression through the phosphatidylinositol 3-kinase/Akt pathway and the Nrf2 transcription factor in response to the antioxidant phytochemical carnosol. J Biol Chem. 2004; 279:8919–29. 10.1074/jbc.M309660200 14688281

[22] Rojo AI, Rada P, Mendiola M, Ortega-Molina A, Wojdyla K, Rogowska-Wrzesinska A, Hardisson D, Serrano M, Cuadrado A. The PTEN/NRF2 axis promotes human carcinogenesis. Antioxid Redox Signal. 2014; 21:2498–514. 10.1089/ars.2014.5843 24892215 PMC4245871

[23] Li FC, Zhang N, Chen WS, Chen QX. Endplate degeneration may be the origination of the vacuum phenomenon in intervertebral discs. Med Hypotheses. 2010; 75:169–71. 10.1016/j.mehy.2010.02.012 20580165

[24] Han Y, Li X, Yan M, Yang M, Wang S, Pan J, Li L, Tan J. Oxidative damage induces apoptosis and promotes calcification in disc cartilage endplate cell through ROS/MAPK/NF-κB pathway: Implications for disc degeneration. Biochem Biophys Res Commun. 2019; 516:1026–32. 10.1016/j.bbrc.2017.03.111 28342871

[25] Kang L, Liu S, Li J, Tian Y, Xue Y, Liu X. Parkin and Nrf2 prevent oxidative stress-induced apoptosis in intervertebral endplate chondrocytes via inducing mitophagy and anti-oxidant defenses. Life Sci. 2020; 243:117244. 10.1016/j.lfs.2019.117244 31891721

[26] Rade M, Määttä JH, Freidin MB, Airaksinen O, Karppinen J, Williams FMK. Vertebral Endplate Defect as Initiating Factor in Intervertebral Disc Degeneration: Strong Association Between Endplate Defect and Disc Degeneration in the General Population. Spine (Phila Pa 1976). 2018; 43:412–9. 10.1097/BRS.0000000000002352 28749857 PMC5756623

[27] Zhong R, Wei F, Wang L, Cui S, Chen N, Liu S, Zou X. The effects of intervertebral disc degeneration combined with osteoporosis on vascularization and microarchitecture of the endplate in rhesus monkeys. Eur Spine J. 2016; 25:2705–15. 10.1007/s00586-016-4593-2 27220969

[28] Ao X, Wang L, Shao Y, Chen X, Zhang J, Chu J, Jiang T, Zhang Z, Huang M. Development and Characterization of a Novel Bipedal Standing Mouse Model of Intervertebral Disc and Facet Joint Degeneration. Clin Orthop Relat Res. 2019; 477:1492–504. 10.1097/CORR.0000000000000712 31094848 PMC6554109

[29] Vergroesen PP, Kingma I, Emanuel KS, Hoogendoorn RJ, Welting TJ, van Royen BJ, van Dieën JH, Smit TH. Mechanics and biology in intervertebral disc degeneration: a vicious circle. Osteoarthritis Cartilage. 2015; 23:1057–70. 10.1016/j.joca.2015.03.028 25827971

[30] Yuan FL, Xu RS, Ye JX, Zhao MD, Ren LJ, Li X. Apoptotic bodies from endplate chondrocytes enhance the oxidative stress-induced mineralization by regulating PPi metabolism. J Cell Mol Med. 2019; 23:3665–75. 10.1111/jcmm.14268 30892812 PMC6484318

[31] Xiao L, Xu X, Zhang F, Wang M, Xu Y, Tang D, Wang J, Qin Y, Liu Y, Tang C, He L, Greka A, Zhou Z, et al. The mitochondria-targeted antioxidant MitoQ ameliorated tubular injury mediated by mitophagy in diabetic kidney disease via Nrf2/PINK1. Redox Biol. 2017; 11:297–311. 10.1016/j.redox.2016.12.022 28033563 PMC5196243

[32] Yao X, Yu S, Jing X, Guo J, Sun K, Guo F, Ye Y. PTEN inhibitor VO-OHpic attenuates GC-associated endothelial progenitor cell dysfunction and osteonecrosis of the femoral head via activating Nrf2 signaling and inhibiting mitochondrial apoptosis pathway. Stem Cell Res Ther. 2020; 11:140. 10.1186/s13287-020-01658-y 32228695 PMC7106818

[33] Wang W, Jing X, Du T, Ren J, Liu X, Chen F, Shao Y, Sun S, Yang G, Cui X. Iron overload promotes intervertebral disc degeneration via inducing oxidative stress and ferroptosis in endplate chondrocytes. Free Radic Biol Med. 2022; 190:234–46. 10.1016/j.freeradbiomed.2022.08.018 35981695

[34] Shao Y, Sun L, Yang G, Wang W, Liu X, Du T, Chen F, Jing X, Cui X. Icariin protects vertebral endplate chondrocytes against apoptosis and degeneration via activating Nrf-2/HO-1 pathway. Front Pharmacol. 2022; 13:937502. 10.3389/fphar.2022.937502 36176424 PMC9513224

